# Xanthine Oxidase-Derived ROS Display a Biphasic Effect on Endothelial Cells Adhesion and FAK Phosphorylation

**DOI:** 10.1155/2016/9346242

**Published:** 2016-07-27

**Authors:** Meriem H. Ben-Mahdi, Pham My-Chan Dang, Marie-Anne Gougerot-Pocidalo, Yvonne O'Dowd, Jamel El-Benna, Catherine Pasquier

**Affiliations:** ^1^Veterinary National Superior School of Algiers, BP 161, Hacène Badi, El Harrach, 16200 Algiers, Algeria; ^2^INSERM-U1149, CNRS-ERL 8252, Centre de Recherche sur l'Inflammation, 75018 Paris, France; ^3^Laboratoire d'Excellence Inflamex, DHU FIRE, Faculté de Médecine, Université Paris Diderot, Sorbonne Paris Cité, Site Xavier Bichat, 75018 Paris, France

## Abstract

In pathological situations such as ischemia-reperfusion and acute respiratory distress syndrome, reactive oxygen species (ROS) are produced by different systems which are involved in endothelial cells injury, ultimately leading to severe organ dysfunctions. The aim of this work was to study the effect of ROS produced by hypoxanthine-xanthine oxidase (Hx-XO) on the adhesion of human umbilical vein endothelial cells (HUVEC) and on the signaling pathways involved. Results show that Hx-XO-derived ROS induced an increase in HUVEC adhesion in the early stages of the process (less than 30 min), followed by a decrease in adhesion in the later stages of the process. Interestingly, Hx-XO-derived ROS induced the same biphasic effect on the phosphorylation of the focal adhesion kinase (FAK), a nonreceptor tyrosine kinase critical for cell adhesion, but not on ERK1/2 phosphorylation. The biphasic effect was not seen with ERK1/2 where a decrease in phosphorylation only was observed. Wortmannin, a PI3-kinase inhibitor, inhibited ROS-induced cell adhesion and FAK phosphorylation. Orthovanadate, a protein tyrosine phosphatase inhibitor, and Resveratrol (Resv), an antioxidant agent, protected FAK and ERK1/2 from dephosphorylation and HUVEC from ROS-induced loss of adhesion. This study shows that ROS could have both stimulatory and inhibitory effects on HUVEC adhesion and FAK phosphorylation and suggests that PI3-kinase and tyrosine phosphatase control these effects.

## 1. Introduction 

In pathological situations such as ischemia-reperfusion and acute respiratory distress syndrome, large amounts of reactive oxygen species (ROS) are produced by different enzymatic systems such as xanthine oxidase, mitochondria, and the phagocyte NADPH oxidase NOX2. These ROS are believed to be involved in endothelial cell injury leading to severe tissue and organ dysfunctions [1, 2].

Oxidative stress, resulting from an imbalance between oxidant production and antioxidant systems, has been reported to induce alterations in signaling pathways leading to modulation of cellular functions, apoptosis, and necrosis [3, 4]. Endothelial cell apoptosis has been described to occur in pathological situations such as acute respiratory distress syndrome, allograft rejection, and atherosclerosis [5, 6]. Loss of cell adhesion to the extracellular matrix results in endothelial cell death [7, 8]. The activity and extent of tyrosine phosphorylation of focal adhesion kinase (FAK) are often used as a hallmark of cell adhesion [9]. FAK is a nonreceptor tyrosine kinase in which phosphorylation and kinase activity are closely regulated by integrin-mediated cell adhesion [10]. FAK may, therefore, play an essential role in integrin signaling and cell survival of anchorage dependent cells [11]. There is increasing evidence that these integrin-induced signals may act together with mitogenic signaling pathways via MAP kinases, in particular extracellular signal regulated protein kinases (ERK1/2), to coordinate cell proliferation and cell survival [12–14].

Phosphorylation and dephosphorylation events play a critical role in the signal transduction pathways that regulate various processes in living cells. ROS have been reported in various cells to alter the phosphorylation of several key proteins involved in signaling pathways [15–17], sometimes with contrasting/conflicting results. However, less is known about the consequences of exposure of cells to oxidative stress over time. The aim of this work is to study the effect of ROS on endothelial cell adhesion to the matrix and the signaling pathways such as FAK and ERK1/2 phosphorylation.

## 2. Materials and Methods 

### 2.1. Reagents

Hank's balanced salt solution, with or without Ca^2+^ and Mg^2+^ (HBSS or HBSS wo), was obtained from GIBCO (Invitrogen, Paisley, UK). Bacto-gelatin was from DIFCO (Detroit, Michigan, USA). Reagents for cell culture were supplied by GIBCO (Invitrogen, Paisley, UK); 100 mm dishes and 6- and 24-well plates were from Costar (Polylabo, Strasbourg, France). Mouse monoclonal FITC-conjugated antihuman factor VIII, mouse monoclonal antibody anti-human focal adhesion kinase (anti-p125FAK), and rabbit polyclonal antibodies anti-human ERK1 and anti-ERK2 were from Santa Cruz Biotechnology (Santa Cruz, CA, USA). Mouse monoclonal antibody anti-human active phosphorylated ERK1/2 was from New England Biolabs (Beverly, MA). Mouse monoclonal anti-phosphotyrosine (anti-Tyr(P)) antibody was from Upstate Biotechnology (Lake Placid, NY). The rainbow markers (high range), sheep anti-mouse IgG conjugated horseradish peroxidase antibody, and ECL Western blot detection system were from Amersham Biosciences (GE Healthcare Europe Gmbh, Orsay, France). Alkaline phosphatase conjugated goat anti-rabbit IgG antibody was from Jackson Laboratories. Nitrocellulose membranes were from Schleicher and Schuell (Dassel, Germany). PD098059, wortmannin, and LY294002 were from Calbiochem (La Jolla, CA, USA). Protein G Sepharose 4 fast flow media were provided by Amersham Biosciences (GE Healthcare Europe Gmbh, Orsay, France). Calcein-acetoxymethyl (calcein-AM) was purchased from Molecular Probes (Invitrogen, Paisley, UK). Resveratrol was kindly donated by Dr. Bagchi (Creighton University School of Pharmacy and Allied Health Professions, Omaha, USA). All other products were from Sigma (St. Louis, Mo, USA).

### 2.2. Endothelial Cell Culture

Endothelial cells from human umbilical cords veins (HUVECs) were harvested by 0.05% collagenase treatment for 15 minutes at 37°C, as previously described [18, 19]. The cells were grown in M199 medium supplemented with fetal calf serum (20%). Cells were incubated at 37°C in humidified atmosphere with 5% CO_2_. At confluence adherent cells were harvested following trypsin-EDTA treatment (0.05% and 0.02%, resp.). Primary-passage HUVECs were seeded into 2% gelatin-coated 100 mm petri dishes and 6- and 24-well tissue culture plates. Culture medium was replaced every 2 days. Adherent cells were identified by their cobblestone appearance at confluence and positive labelling with mouse FITC conjugated antihuman factor VIII. Only cells of secondary cultures were used for the studies.

### 2.3. Exposure of HUVECs to ROS

HUVECs were washed with HBSS and incubated with hypoxanthine (Hx, 2 × 10^−4^ M) and xanthine oxidase (XO, 4.5 mU/mL) for various times (5 to 90 minutes) in HBSS buffer. Hypoxanthine oxidation by xanthine oxidase, generated around 3 nmol/mL/minutes of superoxide anion (O_2_^•−^) and 50 nmol/mL of hydrogen peroxide (H_2_O_2_) over a 30-minute period.

### 2.4. HUVECs Adhesion Assay

Adhesion to the matrix was measured with a Calcein-AM assay as previously described [20]. Following exposure to the different treatments, HUVECs monolayers were thoroughly washed to remove nonadherent cells. Cells adherent to the matrix were loaded with 2.5 *μ*M Calcein-AM and fluorescence was analysed in a FLUOstar plate reader (BMG, Lab Technologies, France) using a 480 nm excitation wavelength and recording of green fluorescence at 520 nm. Values were expressed as mFU units per well. Each experiment was performed in triplicate.

### 2.5. Immunoprecipitation

Control or Hx-XO treated HUVECs in 100 mm petri dishes were lysed by incubation for 30 minutes in 500 *μ*L of ice-cold immunoprecipitation (IP) buffer (10 mM Tris/HCl, pH 7.6, 50 mM NaF, 30 mM sodium pyrophosphate, 1% Triton X-100, 1 mM PMSF, 100 *μ*M orthovanadate, 5 mM EDTA, 50 mM NaCl, 50 *μ*g/mL leupeptin, 10 *μ*g/mL aprotinin, and 250 *μ*g/mL trypsin inhibitor). Samples were sonicated and centrifuged at 15,000 ×g for 10 minutes to remove cellular and nuclear debris. Protein content in clear supernatants was measured using the Bio-Rad protein assay reagent. Samples were incubated with anti-FAK antibody (5 *μ*g/4 × 10^6^ cells) for 2 h at 4°C. Protein was immunoprecipitated using gamma-bind G Sepharose beads overnight at 4°C and beads then were washed three times with cold immunoprecipitation buffer. Sodium dodecyl sulfate (SDS) buffer containing 62.5 mM Tris-HCl pH 6.8, 2.5% SDS, 3% 2 beta-mercaptoethanol, 10% glycerol, and 0.01% bromophenol blue [21] was added and the beads were then boiled for 3 minutes to dissociate the proteins.

### 2.6. Electrophoresis and Western Blot Analysis

Immunoprecipitated proteins or whole cell lysates were subjected to SDS-polyacrylamide gel electrophoresis (PAGE) in 9% polyacrylamide gels using standard techniques [21, 22]. The separated proteins were transferred to nitrocellulose membranes. The membranes were first blocked in 5% nonfat powdered milk/Tris-buffered saline with Tween (TBS-T) (10 mM Tris, 100 mM NaCl, pH 7.5, and 0.1% Tween 20) for 1 hour at room temperature. The membranes were then rinsed once with TBS-T and incubated in 1.5% BSA/TBS-T with anti-phosphotyrosine antibody (1/7500). To ensure that changes in phosphotyrosine content were not attributed to the difference in protein content, immunoprecipitated proteins were incubated in parallel with monoclonal anti-FAK antibody as a control. Similarly, ERK activation was detected by incubating the membranes with an antiactive phosphorylated ERK1/2 antibody (1/5000) overnight at 4°C. After washing, membranes were further incubated with sheep anti-mouse horseradish peroxidase-conjugated secondary antibody (1/15000) for 1 hour, washed, and developed according to the supplier's protocol using ECL detection system and exposed to X-ray film (Hyperfilm MP, Amersham Biosciences, GE Healthcare Europe Gmbh, Orsay, France). The amount of ERK1/2 loaded in each lane was checked by incubation of the same membrane with anti-ERK1 and anti-ERK2 antibodies (1/5000) and detected by an alkaline phosphatase conjugated anti-rabbit IgG antibody. The intensity of the bands was quantified using NIH Image software.

### 2.7. Statistical Analysis

All experiments were performed in triplicate and repeated at least three times. Results are expressed as mean ± standard deviation. Data were compared using analysis of variance (ANOVA) followed by a Fischer test.

## 3. Results 

### 3.1. Effect of ROS on HUVECs Adhesion to the Matrix

**Figure 1:**
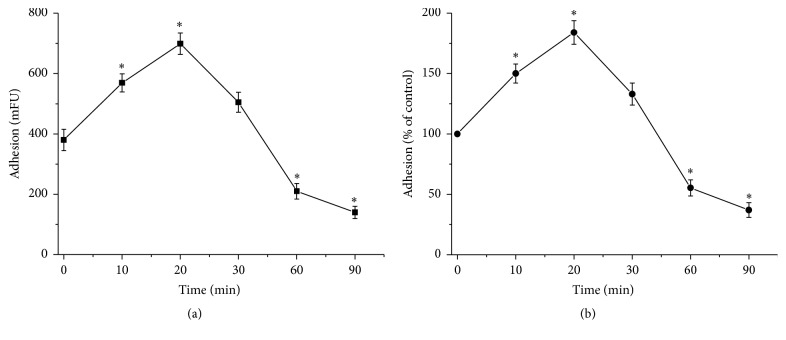
Hx-XO-derived ROS induced time-dependent biphasic HUVEC adhesion. HUVECs monolayers were treated with Hx-XO (4.5 mU/mL) for different time periods and then thoroughly washed to remove nonadherent cells. Cells adherent to the matrix were loaded with 2.5 *μ*M Calcein-AM and fluorescence was analysed in a FLUOstar plate reader (BMG, Lab Technologies, France) using a 480 nm excitation wavelength and recording of green fluorescence at 520 nm. Values were expressed as mFU units per well (a) or as percentage of control cells without Hx-XO (b). Results are expressed as mean ± SD, *n* = 5, ^*∗*^ *P* < 0.01 versus untreated cells.

To determine the consequences of oxidative stress on HUVECs adhesion to the matrix, HUVECs were incubated for 10, 20, 30, 60, or 90 minutes with Hx-XO, a ROS generating system, and adhesion was measured using a Calcein-AM probe. Results show that HUVECs adhesion was significantly increased within the first 20 min of treatment with Hx-XO-derived ROS (Figure 1). Following 30 min of ROS treatment, HUVEC adhesion declined and decreased significantly to 37.5 ± 6.8% of that seen in control untreated HUVECs. The effects of Hx-XO were abrogated by the addition of SOD in the media (data not shown), indicating the role of superoxide anions and derived ROS. These results clearly show that Hx-XO-derived ROS exert an early stimulatory effect and a late inhibitory effect on endothelial cell adhesion.

### 3.2. ROS Induced a Biphasic Effect on FAK Phosphorylation but Not on ERK1/2 Phosphorylation in HUVECs

**Figure 2:**
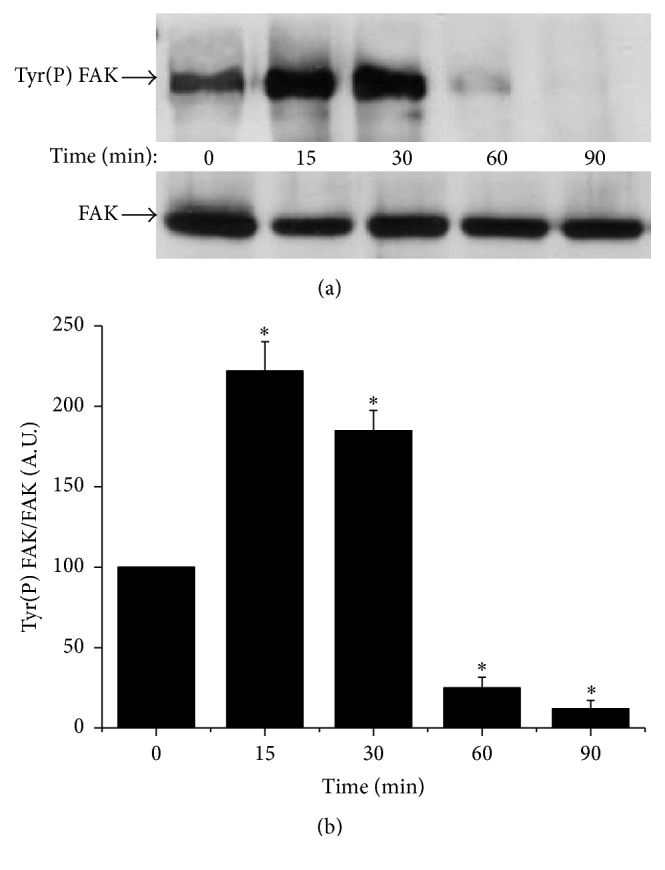
Hx-XO-derived ROS induced time-dependent biphasic FAK tyrosine phosphorylation. HUVECs were treated with Hx-XO (4.5 mU/mL) for the indicated times after which cells were harvested and the soluble fraction analysed for FAK activation. (a) FAK was immunoprecipitated using a monoclonal antibody, and its phosphorylation state and expression were analysed by Western blot (WB) with anti-phosphotyrosine and anti-FAK antibodies. (b) Phosphorylated FAK from different experiments was quantified by densitometry and corrected for the amount of FAK which was quantified by densitometry. Results are expressed as mean ± SEM, *n* = 4. ^*∗*^ *P* < 0.001 versus untreated cells.

**Figure 3:**
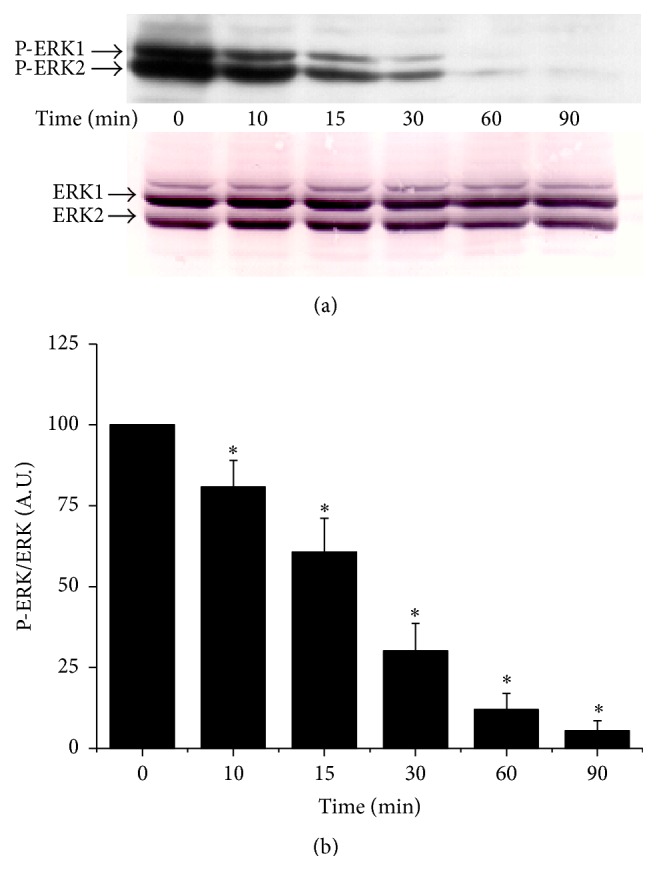
Hx-XO-derived ROS induced time-dependent dephosphorylation of ERK1/2. HUVECs were treated with Hx-XO (4.5 mU/mL) for the indicated times after which cells were harvested and the soluble fraction analysed for ERK1/2 phosphorylation. Western blot analysis with anti-phospho-ERK1/2 was performed as described in Materials and Methods. Total ERK1/2 proteins levels at all times points were equivalent as measured by immunoblot of the same membranes using antibodies against total ERK1/2. (b) Phosphorylated ERK1/2 from different experiments was quantified by densitometry and corrected for the amount of ERK1/2 which was quantified by densitometry. Results are expressed as mean ± SEM, *n* = 4. ^*∗*^ *P* < 0.001 versus untreated cells.

As FAK plays a pivotal role in adhesion mechanisms [7] and ERK1/2 in the cellular survival [12], we studied the effect of Hx-XO-derived ROS on the phosphorylation of both kinases as an indicator of their activation. After immunoprecipitation with an anti-FAK antibody, the immunoprecipitates were probed with anti-phosphotyrosine or anti-FAK antibodies. As shown in Figure 2, Hx-XO treatment induced an early increase in tyrosine phosphorylation of FAK, reaching a maximum at 15 minutes, and then dramatically decreasing thereafter. Immunoblotting with anti-FAK antibody showed that the same amount of FAK protein was loaded in each lane. However, Hx-XO did not induce the early phase of increase of ERK1/2 phosphorylation (Figure 3). A time-dependent dephosphorylation of ERK1/2 was induced resulting in barely detectable levels of phospho-ERK1/2 following 60 minutes and 90 minutes of Hx-XO treatment. Detection of ERK1 and ERK2 with specific antibodies confirmed that the same amounts of ERK1/2 proteins were present in each sample. In conclusion, Hx-XO-derived ROS induced a dual effect on FAK phosphorylation with an early increase followed by a later decrease. This dual effect was not observed with ERK1/2 as only a decrease in phosphorylation was observed.

### 3.3. Wortmannin a PI3-Kinase Inhibitor Inhibits ROS-Induced FAK Phosphorylation and Cells Adhesion

**Figure 4:**
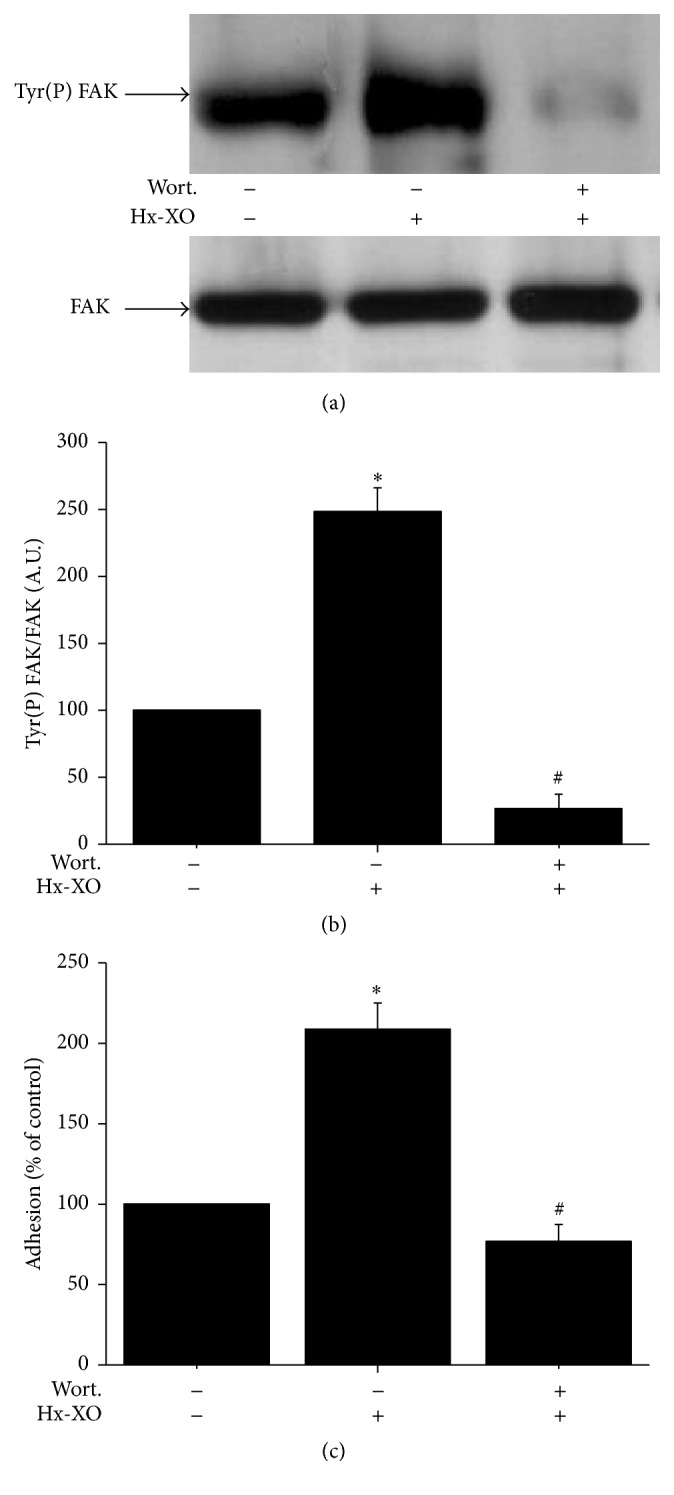
Effect of wortmannin, a PI-3 kinase inhibitor on the early phase of FAK phosphorylation and HUVECs adhesion. HUVECs were preincubated for 20 minutes with PI-3 kinase inhibitor wortmannin (100 nM) and then treated with Hx-XO (4.5 mU/mL) for 15 minutes. (a) FAK was immunoprecipitated using a monoclonal antibody and its phosphorylation state and expression were analysed by Western blot with anti-phosphotyrosine and anti-FAK antibodies. (b) Phosphorylated FAK from different experiments was quantified by densitometry and corrected for the amount of FAK which was quantified by densitometry. (c) HUVECs adhesion was measured after 15 minutes of treatment with Hx-XO by loading cells with 2.5 *μ*M Calcein-AM. Results are expressed as % of control without Hx-XO (mean ± SEM, *n* = 3, ^*∗*^ *P* < 0.001 Hx-XO versus untreated controls; ^#^wortmannin versus Hx-XO alone).

Inhibition of both PI3-kinase activity and the consequent Akt serine phosphorylation accelerated oxidative stress-induced apoptosis [23]. We investigated whether PI3-kinase, which has been shown to bind FAK [24], could participate in the early ROS-induced increase of FAK tyrosine phosphorylation by using wortmannin, a potent PI-3 kinase inhibitor. Results show that wortmannin strongly inhibited a ROS-induced increase of FAK phosphorylation at 15 minutes (Figures 4(a) and 4(b)). This result suggested that under oxidative stress PI-3 kinase could regulate FAK activation. LY294002 (50 *μ*M), another PI3-kinase inhibitor, induced similar effects (data not shown). We then analysed the effect of wortmannin on cell adhesion after ROS treatment. Using Calcein-AM probe, we observed that, after 15 minutes of Hx-XO treatment, adhesion decreased significantly in cells treated with wortmannin (100 nM) compared to cells treated with Hx-XO alone (Figure 4(c)). These results suggest that the “PI3-kinase/FAK-axis” controls the early phase of ROS-mediated HUVEC adhesion.

### 3.4. A Tyrosine Phosphatase Inhibitor Reversed the Late Phase ROS Inhibitory Effect

**Figure 5:**
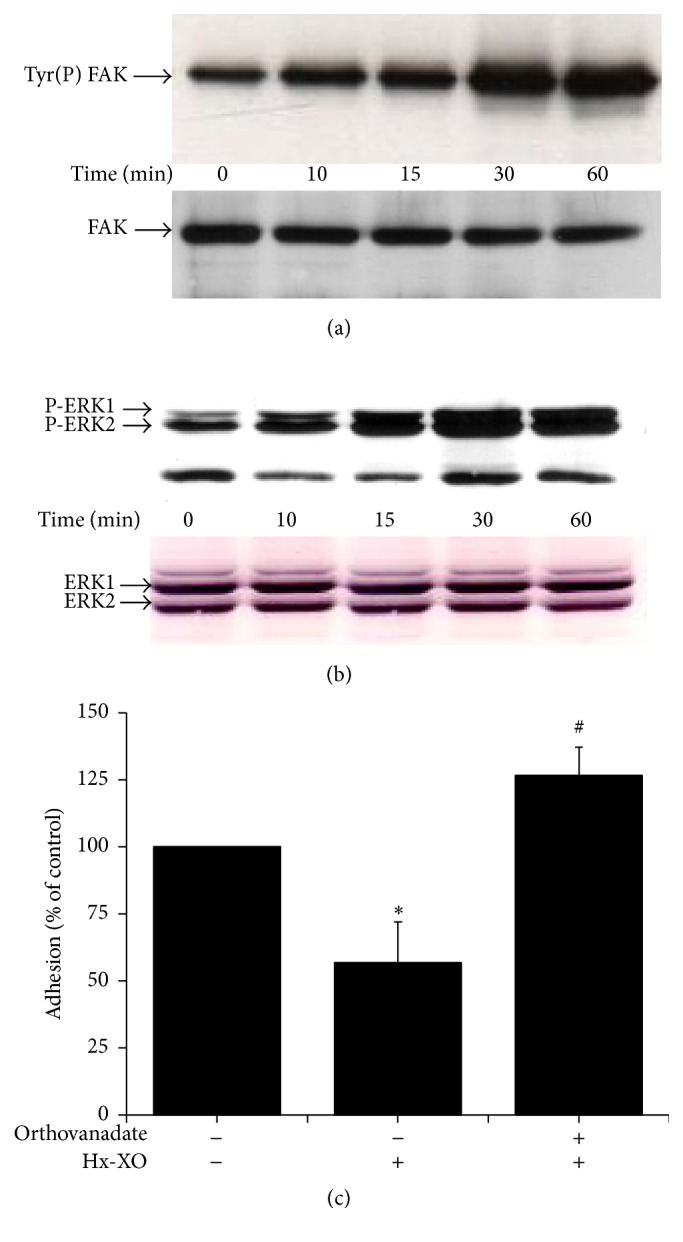
Effect of orthovanadate, a tyrosine phosphatase inhibitor on ROS-induced dephosphorylation of FAK and ERK1/2. HUVECs were preincubated for 30 min with 100 mM of orthovanadate, washed twice, and then treated with Hx-XO (4.5 mU/mL) for the indicated times. (a) The cells were harvested and the soluble fraction was analysed for FAK activation. FAK was immunoprecipitated using a monoclonal antibody, and its phosphorylation state and expression were analysed by Western blot with anti-phosphotyrosine and anti-FAK antibodies. (b) Cells were harvested and the soluble fraction was analysed by Western blot with anti-phospho-ERK1/2. Total ERK1/2 proteins levels at all times points were equivalent as measured by immunoblot of the same membranes using antibodies against total ERK1/2 coupled to alkaline phosphatase substrate. (c) HUVECs adhesion was determined by loading cells with Calcein-AM as described in Materials and Methods after 60 minutes of Hx-XO treatment. Results are expressed as % of control without Hx-XO (mean ± SEM, *n* = 3. ^*∗*^ *P* < 0.001 Hx-XO versus untreated controls; ^#^orthovanadate versus Hx-XO alone).

The dephosphorylation of FAK and ERK1/2 in the late phase of ROS treatment suggests the involvement of a phosphatase which could be activated by a long-term ROS treatment. We then assessed the effect of orthovanadate, a tyrosine phosphatase inhibitor, on ROS-induced dephosphorylation of FAK and ERK1/2. Results show that incubation of HUVECs with 100 mM orthovanadate prior to Hx-XO restored and even increased the level of FAK and ERK1/2 phosphorylation in HUVECs (Figures 5(a) and 5(b)). Quantification analysis showed that, in the presence of orthovanadate and 60 min of ROS treatment, phosphorylation of FAK and ERK1/2 reached 257.5 ± 26.8% and 216.9 ± 15.5% of that seen with control untreated HUVECs respectively (mean ± SEM; *n* = 3, *P* = 0.05). Orthovanadate also reversed the ROS-inhibitory effect on cell adhesion mainly at 60 min (Figure 5(c)). These results suggested the involvement of a ROS-induced activation of protein tyrosine phosphatase(s) involved in the ROS-induced dephosphorylation of ERK1/2 and FAK and ROS-induced inhibition of HUVEC adhesion.

### 3.5. The Antioxidant Agent Resveratrol Prevents ROS-Induced Loss of Adhesion and FAK/ERK Dephosphorylation

**Figure 6:**
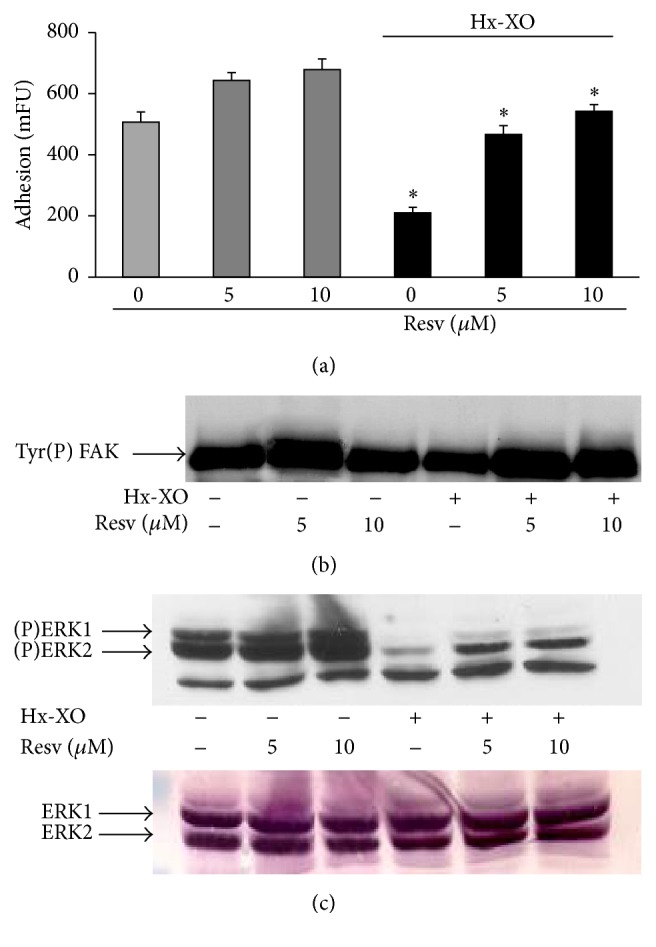
Resveratrol protected HUVEC from ROS-induced loss of adhesion and from FAK/ERK dephosphorylation. Cells were preincubated with 5 and 10 *μ*M of Resveratrol for 4 hours and then treated with Hx-XO (4.5 mU/mL) for 60 minutes. (a) Adherent HUVECs were determined by loading cells with 2.5 *μ*M Calcein-AM as described in Materials and Methods. (b) The cells were harvested and the soluble fraction was analysed for FAK activation. FAK was immunoprecipitated using a monoclonal antibody, and its phosphorylation state was analysed by Western blot with anti-phosphotyrosine. (c) Cells were harvested 30 minutes later for analysis of ERK1/2 activity by Western blot with anti-phospho-ERK1/2 antibodies. Results are expressed as mean ± SD of three experiments, each performed in triplicate. ^*∗*^ *P* < 0.001 compared to the Hx-XO alone.

To verify that HUVECs alterations were due to oxidative injury caused by oxidative stress induced by the Hx-XO system, HUVECs were pretreated with the antioxidant molecule, trans-3,4′,5-trihydroxystilbene (Resveratrol) [25, 26]. Results show that Resveratrol by itself increased HUVEC adhesion (Figure 6(a)). Treatment of cells by Hx-XO for 60 min resulted in the inhibition of cell adhesion as expected. Resveratrol at 5 and 10 *μ*M significantly protected the cells from ROS. Under these experimental conditions, Resveratrol protected from Hx-XO-induced FAK and ERK1/2 dephosphorylation (Figures 6(b) and 6(c)). Quantification analysis showed that, in the presence of 10 *μ*M Resveratrol and after ROS treatment, phosphorylated FAK and ERK2 are 187.5 ± 16.8% and 166.9 ± 11.5% of control HUVECs with Hx-XO alone, respectively (mean ± SEM; *n* = 3, *P* = 0.05).

## 4. Discussion 

In this study, we have demonstrated that oxidative stress, produced by the hypoxanthine-xanthine oxidase (Hx-XO) system, modulated both HUVECs adhesion and FAK phosphorylation. During the first twenty minutes of ROS exposure, HUVECs adhesion was significantly increased in parallel with FAK phosphorylation. This early ROS-induced adhesion and FAK phosphorylation were dependent on PI3-kinase. Later, we observed a progressive decrease in cell adhesion, concomitant with a decreased in FAK and ERK1/2 phosphorylation. These latter dephosphorylation events could be related to the ROS-induced activation of one or several tyrosine phosphatases. These events are prevented by the antioxidant agent Resveratrol. These results demonstrated that ROS can have dual contrasting effects on HUVECs adhesion to the matrix depending on the length of exposure. This phenomenon is critical for HUVEC functioning and survival.

ROS induced a biphasic effect on cell adhesion and FAK phosphorylation, following a transient increase during the first 20 minutes, a dephosphorylation of FAK and a decrease of cell adhesion occurred in a time-dependent manner. The early ROS-induced increase of FAK phosphorylation was regulated by PI3-kinase as shown by its inhibition by wortmannin. These results suggest that ROS can modulate PI3-kinase directly or indirectly as previously described [27, 28]. The increase in FAK phosphorylation was parallel to the increase of HUVECs adhesion, suggesting a critical role of this kinase in preservation of cellular adhesion induced by ROS. FAK dephosphorylation under sustained oxidative stress as observed after 30 minutes of Hx-XO treatment was associated with a loss of HUVECs adhesion to the matrix. Several reports described the role played by FAK phosphorylation in promoting cell survival [7, 29–33]. In fact, microinjection of anti-FAK antibody or treatment with competitive peptide for the catalytic site of FAK, induce cell apoptosis [11, 32]. Sonoda et al. suggested that FAK is upstream of the PI3-kinase-Akt survival-pathway in hydrogen peroxide-induced apoptosis of human glioblastoma cells T98G [23]. The inhibition of FAK tyrosine phosphorylation by wortmannin suggests that FAK activation under our experimental conditions of oxidative stress is downstream of PI3-kinase activity in endothelial cells.

We also showed the involvement of tyrosine phosphatases in the oxidative stress regulation of these kinases. The very quick-acting dephosphorylation of ERK1/2 suggests the activation of constitutive phosphatases by ROS [34–36]. Dephosphorylation of activated ERK1/2 may be mediated by PP2A (protein phosphatase 2A) or by a family of recently described MAPK phosphatases (MKP) with dual specificity (Thr/Tyr). This family includes MKPs 1–4 [37, 38], which are inducible by cellular stress and rapidly translocate to the nucleus [39–41]. As ROS-induced FAK and ERK1/2 dephosphorylation differed in their kinetics under oxidative stress, it could be hypothesized that ROS induced the activation of different phosphatases possibly involved in these processes.

The protective effect of polyphenolic antioxidant agent such as Resveratrol [25, 26], on the ROS-induced decrease in HUVECs adhesion and ERK1/2 dephosphorylation, confirmed the role of ROS in modulating transductional signals and therefore regulating cellular functions.

In our model of time-dependent oxidative stress we propose that initially the ROS-induced increase of FAK tyrosine phosphorylation is dependent on PI3-kinase. This leads to continued HUVECs adhesion in spite of a rapid dephosphorylation of ERK1/2. In the late phase of this process, ROS-induced activation of different phosphatases may be involved in ERK1/2 as well as FAK dephosphorylation, leading to a decrease in HUVECs adhesion. Kinetic studies should be performed to further investigate the effect of ROS on cell function.

In conclusion, our data has demonstrated a time-dependent biphasic effect of ROS on FAK phosphorylation and endothelial cells adhesion. Under these conditions, FAK phosphorylation plays a critical role in regulating cell adhesion to the matrix and was dependent on equilibrium between PI3-kinase and protein tyrosine phosphatases. Such mechanisms could occur during different pathological inflammatory conditions as a result of the release of ROS in proximity to endothelial cells.

## Abbreviations

Anti-Tyr(P): Anti-phosphotyrosine antibody
ERK: Extracellular signal regulated kinase
FAK: Focal adhesion kinase
Hx: Hypoxanthine
IP: Immunoprecipitation
Resv: Resveratrol
ROS: Reactive oxygen species
WB: Western blot
XO: Xanthine oxidase.

## References

[1] Granger D. N., Benoit J. N., Suzuki M., Grisham M. B. (1989). Leukocyte adherence to venular endothelium during ischemia-reperfusion. *American Journal of Physiology-Gastrointestinal and Liver Physiology*.

[2] Suzuki M., Inauen W., Kvietys P. R. (1989). Superoxide mediates reperfusion-induced leukocyte-endothelial cell interactions. *American Journal of Physiology—Heart and Circulatory Physiology*.

[3] Burdon R. H. (1996). Control of cell proliferation by reactive oxygen species. *Biochemical Society Transactions*.

[4] Davies K. J. A. (1999). The broad spectrum of responses to oxidants in proliferating cells: a new paradigm for oxidative stress. *IUBMB Life*.

[5] Polunovsky V. A., Chen B., Henke C. (1993). Role of mesenchymal cell death in lung remodeling after injury. *The Journal of Clinical Investigation*.

[6] Cai W.-J., Devaux B., Schaper W., Schaper J. (1997). The role of Fas/APO 1 and apoptosis in the development of human atherosclerotic lesions. *Atherosclerosis*.

[7] Frisch S. M., Vuori K., Ruoslahti E., Chan-Hui P.-Y. (1996). Control of adhesion-dependent cell survival by focal adhesion kinase. *Journal of Cell Biology*.

[8] Hoyt D. G., Mannix R. J., Gerritsen M. E., Watkins S. C., Lazo J. S., Pitt B. R. (1996). Integrins inhibit LPS-induced DNA strand breakage in cultured lung endothelial cells. *American Journal of Physiology-Lung Cellular and Molecular Physiology*.

[9] Lin T. H., Aplin A. E., Shen Y. (1997). Integrin-mediated activation of MAP kinase is independent of FAK: evidence for dual integrin signaling pathways in fibroblasts. *The Journal of Cell Biology*.

[10] Guan J.-L., Shalloway D. (1992). Regulation of focal adhesion-associated protein tyrosine kinase by both cellular adhesion and oncogenic transformation. *Nature*.

[11] Hungerford J. E., Compton M. T., Matter M. L., Hoffstrom B. G., Otey C. A. (1996). Inhibition of pp125FAK in cultured fibroblasts results in apoptosis. *Journal of Cell Biology*.

[12] Xia Z., Dickens M., Raingeaud J., Davis R. J., Greenberg M. E. (1995). Opposing effects of ERK and JNK-p38 MAP kinases on apoptosis. *Science*.

[13] Le Gall M., Chambard J.-C., Breittmayer J.-P., Grall D., Pouysségur J., Van Obberghen-Schilling E. (2000). The p42/p44 MAP kinase pathway prevents apoptosis induced by anchorage and serum removal. *Molecular Biology of the Cell*.

[14] Seger R., Krebs E. G. (1995). The MAPK signaling cascade. *FASEB Journal*.

[15] Schieven G. L., Kirihara J. M., Burg D. L., Geahlen R. L., Ledbetter J. A. (1993). p72syk tyrosine kinase is activated by oxidizing conditions that induce lymphocyte tyrosine phosphorylation and Ca^2+^ signals. *The Journal of Biological Chemistry*.

[16] Qin S., Inazu T., Takata M., Kurosaki T., Homma Y., Yamamura H. (1996). Cooperation of tyrosine kinases p72syk and p53/56lyn regulates calcium mobilization in chicken B cell oxidant stress signaling. *European Journal of Biochemistry*.

[17] Suzuki Y., Ohsugi K., Ono Y. (1996). Oxidative stress triggers tyrosine phosphorylation in B cells through a redox- and inflammatory cytokine-sensitive mechanism. *Immunology*.

[18] Jaffe E. A., Nachman R. L., Becker C. G., Minick C. R. (1973). Culture of human endothelial cells derived from umbilical veins. Identification by morphologic and immunologic criteria. *The Journal of Clinical Investigation*.

[19] Sellak H., Franzini E., Hakim J., Pasquier C. (1994). Reactive oxygen species rapidly increase endothelial ICAM-1 ability to bind neutrophils without detectable upregulation. *Blood*.

[20] Ben-Mahdi M. H., Gozin A., Driss F., Andrieu V., Christen M.-O., Pasquier C. (2000). Anethole dithiolethione regulates oxidant-induced tyrosine kinase activation in endothelial cells. *Antioxidants and Redox Signaling*.

[21] Belambri S. A., Dang P. M.-C., El-Benna J. (2014). Evaluation of p47phox phosphorylation in human neutrophils using phospho-specific antibodies. *Methods in Molecular Biology*.

[22] Towbin H., Staehelin T., Gordon J. (1979). Electrophoretic transfer of proteins from polyacrylamide gels to nitrocellulose sheets: procedure and some applications. *Proceedings of the National Academy of Sciences of the United States of America*.

[23] Sonoda Y., Watanabe S., Matsumoto Y., Aizu-Yokota E., Kasahara T. (1999). FAK is the upstream signal protein of the phosphatidylinositol 3-kinase-Akt survival pathway in hydrogen peroxide-induced apoptosis of a human glioblastoma cell line. *The Journal of Biological Chemistry*.

[24] Chen H.-C., Appeddu P. A., Isoda H., Guan J.-L. (1996). Phosphorylation of tyrosine 397 in focal adhesion kinase is required for binding phosphatidylinositol 3-kinase. *Journal of Biological Chemistry*.

[25] Frankel E. N., Waterhouse A. L., Kinsella J. E. (1993). Inhibition of human LDL oxidation by resveratrol. *The Lancet*.

[26] Rotondo S., Rajtar G., Manarini S. (1998). Effect of trans-resveratrol, a natural polyphenolic compound, on human polymorphonuclear leukocyte function. *British Journal of Pharmacology*.

[27] Nguyen K. T., Zong C. S., Uttamsingh S. (2002). The role of phosphatidylinositol 3-kinase, rho family GTPases, and STAT3 in ros-induced cell transformation. *Journal of Biological Chemistry*.

[28] Cuda G., Paternò R., Ceravolo R. (2002). Protection of human endothelial cells from oxidative stress: role of Ras-ERK1/2 signaling. *Circulation*.

[29] Sonoda Y., Kasahara T., Yokota-Aizu E., Ueno M., Watanabe S. (1997). A suppressive role of p125FAK protein tyrosine kinase in hydrogen peroxide-induced apoptosis of T98G cells. *Biochemical and Biophysical Research Communications*.

[30] Sonoda Y., Aiba N., Utsubo R., Koguchi E., Hasegawa M., Kasahara T. (2004). Induction of antioxidant enzymes by FAK in a human leukemic cell line, HL-60. *Biochimica et Biophysica Acta (BBA)—Molecular and Cell Biology of Lipids*.

[31] Ilić D., Almeida E. A. C., Schlaepfer D. D., Dazin P., Aizawa S., Damsky C. H. (1998). Extracellular matrix survival signals transduced by focal adhesion kinase suppress p53-mediated apoptosis. *Journal of Cell Biology*.

[32] Mian M. F., Kang C., Lee S. (2008). Cleavage of focal adhesion kinase is an early marker and modulator of oxidative stress-induced apoptosis. *Chemico-Biological Interactions*.

[33] Chan P.-C., Lai J.-F., Cheng C.-H., Tang M.-J., Chiu C.-C., Chen H.-C. (1999). Suppression of ultraviolet irradiation-induced apoptosis by overexpression of focal adhesion kinase in Madin-Darby canine kidney cells. *The Journal of Biological Chemistry*.

[34] Lewis T., Groom L. A., Sneddon A. A., Smythe C., Keyse S. M. (1995). XCL100, an inducible nuclear MAP kinase phosphatase from Xenopus laevis: its role in MAP kinase inactivation in differentiated cells and its expression during early development. *Journal of Cell Science*.

[35] Fischer E. H., Charbonneau H., Tonks N. K. (1991). Protein tyrosine phosphatases: a diverse family of intracellular and transmembrane enzymes. *Science*.

[36] Alessi D. R., Gomez N., Moorhead G., Lewis T., Keyse S. M., Cohen P. (1995). Inactivation of p42 MAP kinase by protein phosphatase 2A and a protein tyrosine phosphatase, but not CL100, in various cell lines. *Current Biology*.

[37] Chu Y., Solski P. A., Khosravi-Far R., Der C. J., Kelly K. (1996). The mitogen-activated protein kinase phosphatases PAC1, MKP-1, and MKP-2 have unique substrate specificities and reduced activity *in vivo* toward the ERK2 sevenmaker mutation. *The Journal of Biological Chemistry*.

[38] Muda M., Boschert U., Dickinson R. (1996). MKP-3, a novel cytosolic protein-tyrosine phosphatase that exemplifies a new class of mitogen-activated protein kinase phosphatase. *The Journal of Biological Chemistry*.

[39] Keyse S. M. (1995). An emerging family of dual specificity MAP kinase phosphatases. *Biochim Biophys Acta*.

[40] Keyse S. M. (2000). Protein phosphatases and the regulation of mitogen-activated protein kinase signalling. *Current Opinion in Cell Biology*.

[41] Keyse S. M. (2008). Dual-specificity MAP kinase phosphatases (MKPs) and cancer. *Cancer and Metastasis Reviews*.

